# Pairwise joint modeling of clustered and high-dimensional outcomes with covariate missingness in pediatric pneumonia care

**DOI:** 10.1002/pst.2197

**Published:** 2022-02-24

**Authors:** Susan Gachau, Edmund Njeru Njagi, Geert Molenberghs, Nelson Owuor, Rachel Sarguta, Mike English, Philip Ayieko

**Affiliations:** 1 Health Services Unit, Kenya Medical Research Institute-Wellcome Trust Research Programme, Nairobi, Kenya; 2 School of Mathematics, University of Nairobi, Nairobi, Kenya; 3 Department of Non-Communicable Disease Epidemiology, London School of Hygiene and Tropical Medicine, London, UK; 4 Center for Statistics, Universiteit Hasselt, Hasselt, Belgium; 5 Interuniversity Institute for Biostatistics and Statistical Bioinformatics, Katholieke Universiteit, Leuven, Belgium; 6 Nuffield Department of Medicine, University of Oxford, Oxford, UK; 7 Department of Infectious Disease Epidemiology, London School of Hygiene and Tropical Medicine, London, UK; 8 Mwanza Intervention Trials Unit, Mwanza, Tanzania

**Keywords:** multiple imputation, pairwise joint modeling, pediatric care, pneumonia, pseudo-likelihood

## Abstract

Multiple outcomes reflecting different aspects of routine care are a common phenomenon in health care research. A common approach of handling such outcomes is multiple univariate analyses, an approach which does not allow for answering research questions pertaining to joint inference. In this study, we sought to study associations among nine pediatric pneumonia care outcomes spanning assessment, diagnosis and treatment domains of care, while circumventing the computational challenge posed by their clustered and high-dimensional nature and incompletely recorded covariates. We analyzed data from a cluster randomized trial conducted in 12 Kenyan hospitals. There were varying degrees of missingness in the covariates of interest, and these were multiply imputed using latent normal joint modeling. We used the pairwise joint modeling strategy to fit a correlated random effects joint model for the nine outcomes. This entailed fitting 36 bivariate generalized linear mixed models and deriving inference for the joint model using pseudo-likelihood theory. We also analyzed the nine outcomes separately before and after multiple imputation. We observed joint effects of patient-, clinician- and hospital-level factors on pneumonia care indicators before and after multiple imputation of missing covariates. In both pairwise joint modeling and separate univariate analysis methods, enhanced audit and feedback improved documentation and adherence to recommended clinical guidelines over time in six and five pneumonia care indicators, respectively. Additionally, multiple imputation improved precision of parameter estimates compared to complete case analysis. The strength and direction of association among pneumonia outcomes varied within and across the three domains of pneumonia care

## 1 Introduction

Multiple responses reflecting different aspects of patients’ care are a common phenomenon in routine care studies, investigating research questions such as the level of adherence to standard quality of care guidelines by clinicians in different health care facilities. Besides complexities associated with multiple outcomes spanning several domains of quality care, routine data are prone to missing information which can occur at patient-, clinician- and/or facility-level.

Despite measuring, for each patient, a correlated vector of response variables, inferences in most routine care studies are based on one primary outcome or multiple separate analyses.^1–3^ Alternatively, the outcomes are combined into a single composite score,^4–7^ to provide global trends and insight into the quality of patient care. While these approaches are relatively straight forward, some research questions require joint modeling of all outcomes simultaneously,^8,9^ for instance, when the association among outcomes and joint effects of covariates on all outcomes are of primary research interest.^8–11^

In principle, a joint model links two or more models, using random effects that capture association among outcomes of interest. Statistically, joint modeling has advantages over separate analyses of multiple outcomes. This includes efficiency gain and bias reduction, especially when data are missing at random (MAR) in some of the outcomes.^8,12–16^ In addition, joint modeling allows for different types of models for the different outcomes^17^ (e.g., linear, non-linear, and generalized linear mixed models), while the interpretation of parameter estimates is the same as interpretation from the separate univariate models.^13^

Although joint models have been extended from the common bivariate to the multivariate cases,^14^ standard fitting procedures are difficult to implement with high-dimensional outcomes.^8,14,16,18–21^ The computational complexities stem from an increase in the number of parameters to be estimated, for every new outcome added into the joint model,^8^ and relatedly the increasing dimension of the random-effects vector.

To overcome these challenges, the shared random-effects model, which assumes that all outcomes share the same set of random effects, can be considered. In this case, the dimension of the random effects does not increase with an increase with the number of outcomes.^8,20^ The price to pay is a sometimes restrictive, less realistic model.^8,16^ For instance, when dealing with discrete outcomes (e.g., binomial and Poisson), that have a natural link between the mean and variance.

A plausible alternative is the pairwise joint modeling approach, which allows fitting of the correlated random-effects joint model, while circumventing the computational complexity associated with a full joint multivariate model.^8,9,11,14^

As mentioned earlier, missing data in either outcomes or covariates is a common problem in routine data. Although joint modeling can be used to mitigate the effect of missing data among outcomes, appropriate strategies of handling missing covariates in high-dimensional joint modeling is hardly addressed in the literature. For instance, a previous joint modeling study reported deletion of case records with missing covariates to alleviate computational challenges.^22^ The repercussion of suboptimal missing data handling techniques include risk of biased and inefficient estimates, hence misleading inferences.^23^

In the present study, we sought to jointly analyze nine binary outcomes, at the same time accounting for covariate missingness in a pediatric routine data set, from a cluster randomized trial conducted in Kenyan hospitals. Specifically, we used multiple imputation, based on the joint modeling (JM) framework to address missing covariates across two levels of the hierarchy. Thereafter, we used the pairwise approach within the pseudo-likelihood framework to estimate the joint effects of covariates on outcomes. This was in addition to estimating the strength of association among nine pneumonia outcomes. Besides joint modeling, we analyzed the nine binary outcomes separately under complete case analysis and after multiple imputation of missing covariates.

The remainder of this article is organized as follows. Section 2 introduces the joint modeling approach using mixed models and the pairwise fitting approach. Section 3 introduces the pneumonia trial data while Section 4 present multilevel multiple imputation model, univariate random effect model and pairwise joint model for pneumonia trial data set. Results under complete case analysis and after multiple imputation are presented in Section 5 and we conclude with a discussion in Section 6.

## 2 Correlated Random-Effects Joint Model

Let *Y_rij_* denote the *r^th^* (*r* = 1,2,…,*p*) outcome for the *i^th^* (*i* = 1,2, …,N) subject in cluster *j* (*j* = 1,2, …, *n_i_*). The corresponding univariate random effects model for the *r^th^* outcome can be defined as

(1) $$h^{-1}\left(E\left(Y_{rij}|{\text{b}}_{\text{ri}},{\text{X}}_{\text{rij}},{\text{Z}}_{\text{rij}}\right)\right)={{\text{X}}^{\prime }}_{rij}\beta_{r}+{{\text{Z}}^{\prime }}_{rij}{\text{b}}_{ri},$$

where *h*^–1^(·) is an appropriate link function depending on the type of outcome (i.e., whether continuous, binary, count, etc.),^12^ ***X_rij_*** is a vector of known covariates with fixed effects ***β_r_***, and ***Z_rij_*** is a vector of covariates with random effects ***b_ri_***. The univariate random effects model can be extended to jointly model all the outcomes simultaneously, by imposing a joint multivariate distribution on the random effects.^14,15,17^ Moreover, the number of random effects can vary among the outcomes of interest. Conditional on the vector of random effects (***b_ri_***), the outcomes are assumed to be independent^8^ and the corresponding log-likelihood contribution for subject *i* equals

(2) $$l_{i}\left(y_{1i},y_{2i},\ldots ,y_{pi}|\Theta^{*}\right)=\text{ln}\int \prod_{r=1}^{p}f_{ri}\left(y_{ri}|{\text{b}}_{\text{ri}},\theta_{r}\right)f\left({\text{b}}_{\text{i}}|\text{D}\right)d{\text{b}}_{\text{i}}.$$

The vector Θ* contains all parameters of the full joint model (i.e., fixed parameters denoted by β* and covariance parameters denoted by Σ*), while *f _ri_*(***y_ri_***|***b_ri_,θ_r_***) is the density of ***y_ri_*** conditional on the random effects for the *r^th^* outcome on subject *i*. The vector of random-effects ***b_i_*** is assumed to follow a multivariate normal distribution with mean zero and covariance matrix ***D***, that is,

$${\text{b}}_{i}=\left(\begin{array}{c} {\text{b}}_{1i}\\ {\text{b}}_{2i}\\ \vdots \\ {\text{b}}_{pi} \end{array}\right)∼N[\left(\begin{array}{c} 0\\ 0\\ \vdots \\ 0 \end{array}\right),\left(\begin{array}{cccc} {\text{D}}_{11} & {\text{D}}_{12} & \cdots & {\text{D}}_{1p}\\ {\text{D}}_{21} & {\text{D}}_{22} & \cdots & {\text{D}}_{2p}\\ \vdots & \vdots & \ddots & \vdots \\ {\text{D}}_{p1} & {\text{D}}_{p2} & \cdots & {\text{D}}_{pp} \end{array}\right)].$$

The elements ***D_rs_*** in ***D*** correspond to blocks of random effects variance–covariance between the *r^th^* and the *s^th^* outcomes (***r, s*** = 1,2,…,*p*). For example, assuming that each outcome has a random intercept (*b*_0_) and a random slope (*b*_1_), then ***D_rs_*** is given by

$${\text{D}}_{rs}=\left[\begin{array}{cccc} \sigma_{b_{0r}}^{2} & \sigma_{b_{0r}}b_{1r} & \sigma_{b_{0r}}b_{0s} & \sigma_{b_{0r}b_{1s}}\\ \; & \sigma_{b_{1r}}^{2} & \sigma_{b_{1r}b_{0s}} & \sigma_{b_{1r}b_{1s}}\\ \; & \; & \sigma_{b_{0s}}^{2} & \sigma_{b_{0s}b_{1s}}\\ \; & \; & \; & \sigma_{b_{1s}}^{2} \end{array}\right].$$

The elements of the variance covariance matrix ***D*** can be used to measure the strength of association between any two outcomes of interest. As mentioned earlier, the dimension of the random effects vector ***b_i_*** in the full joint model, increases with an in increase in the number of outcomes. This leads to computational challenges for high dimensional vectors of outcomes.^8,10,14^

### 2.1 The pairwise modeling approach

In light of computational challenges highlighted above, Fieuws and Verbeke^14^ proposed a pairwise approach within the pseudo-likelihood framework to handle high-dimensional vectors of outcomes. With a vector of *p* outcomes, the pairwise approach maximizes the likelihood for all *Q* = *p*(*p* – 1)/2 pairwise models separately, instead of maximizing the full joint multivariate likelihood.^14,24^ Precisely, this produces a so-called pseudo-likelihood (*pl*) of the following form:

(3) $$pl\left(\Theta \right)=l\left(Y_{1},Y_{2}|\Theta_{12}\right)l\left(Y_{1},Y_{3}|\Theta_{13}\right),\ldots ,l\left(Y_{p-1},Y_{p}|\Theta_{p-1p}\right)=\prod_{r=1}^{p-1}\prod_{s=1}^{p}l\left(Y_{r},Y_{s}|\Theta_{rs}\right).$$

For a given pair of responses (***r, s*** = 1,2..,*p*),*l*(*Y_r_, Y_s_*|***Θ_rs_***) denotes the likelihood, while ***Θ_rs_*** is the vector of all parameters encountered in a pairwise joint model.^14^ The corresponding pseudo-log likelihood function (*pll*) is given by

$$\begin{array}{ll} pll\left(\Theta \right) & =\sum_{r=1}^{p-1}\sum_{s=r+1}^{p}ll\left(Y_{r},Y_{s}|\Theta_{\text{rs}}\right),\\ \; & =\sum_{q=1}^{Q}ll\left({\text{Y}}_{\text{q}}|\Theta_{\text{q}}\right), \end{array}$$

where ***Y_q_*** and ***Θ_q_*** contain all the observations and parameters, respectively, in the *q^th^* response pair (*q* = 1,2,…, *Q*). All Q pair-specific parameter vectors ***Θ_q_*** (*q* = 1,2,…, *Q*) are stacked together into ***Θ*** with fixed parameters denoted by ***β***. It is clear that if ${\hat{\Theta }}_{\text{q}}$ maximizes *l*(*Y_q_*|***Θ_q_***), then $\hat{\Theta }$, the stacked vector combining all ${\hat{\Theta }}_{\text{q}}$, maximizes *pll*(***Θ***).^24^ The asymptotic distribution of $\hat{\Theta }$ is multivariate normal given by

(4) $$\sqrt{N}\left(\hat{\Theta }-\Theta \right)∼MVN\left(0,{\text{H}}^{-1}{\text{GH}}^{-1}\right),$$

where ***H***^−1^***GH***^−1^ is a sandwich estimator and ***H*** and ***G*** are based on cluster-wise Hessians and gradients of the log-pseudo-likelihood function, respectively.^8,10,18,24^ The vector of all parameters in the full joint model (Θ*) and stacked vector from pairwise models (***Θ***) are not equivalent. Specifically, some parameters in Θ* have a single counterpart in ***Θ***, while other elements in Θ* have multiple counterparts in ***Θ***.^8^ A set of fixed effects (β*), for the full joint model, are obtained by averaging duplicate parameter estimates from the pairwise joint models.^8,14^ This can be achieved by multiplying the stacked vector of regression parameters (***β***) with an appropriate weight matrix ***A*** as below

(5) $$\beta^{*}=A\beta .$$

The standard errors follow as the square root of diagonal elements of variance–covariance estimator

(6) $$\Sigma^{*}=\text{A}\left({\text{H}}^{-1}{\text{GH}}^{-1}\right){\text{A}}^{T}.$$

Further details on estimation of fixed effects and corresponding standard errors are presented in the application section.

## 3 Pneumonia Trial Data

In this study, we analyzed routine pediatric data collected in a cluster randomized trial in 12 Kenyan hospitals between March and November 2016.^2,25^ The trial’s objective was to investigate the level of uptake of pediatric pneumonia treatment guidelines recommended by the World Health Organization (WHO) in 2013.^26^ Details on the trial are contained in the trial report.^2^ In brief, hospitals were randomly allocated to the intervention arm or control arm. Six hospitals in the intervention received an enhanced monthly audit and feedback (A&F) report on assessment, diagnosis and treatment of pneumonia cases, a bi-monthly standard A&F report assessing performance and adherence to general inpatient pediatric care guidelines at facility level. Besides A&F reports, the trial intervention package contained network intervention strategies such as peer learning among clinicians across study facilities, workshops and follow-up emails and phone calls by the trial pediatrician. On the other hand, six control hospitals received a bi-monthly standard A&F report and network intervention strategies.^2,25^

During the trial period, 2299 children aged 2 to 59 months were admitted in general pediatric wards with childhood pneumonia in 12 study hospitals. However, this analysis excluded 172/2299 (7.5%) case records lacking admitting clinician’s information. The remaining 2127/2299 (92.5%) patients were admitted by 378 clinicians. Of the 2127 pneumonia cases, 953 (44.8%) were admitted to six intervention hospitals. On average, there were 32 clinicians per hospital, and the number of patients per clinician ranged between 3 and 46. Data were extracted by trained data clerks from pediatric admission record (PAR) (a structured paper based medical record/form used in pediatric wards in CIN hospitals) after discharge from hospital. The data were entered into an open source data capture tool (Research Electronic Data Capture, [REDCap])^27^ using a standard operating procedure manual.

### 3.1 Pediatric pneumonia care indicators

The outcomes of interest were nine pneumonia care indicators spanning three domains of care (Table 1). These are 1 = cough, 2 = difficult breathing, 3 = respiratory rate, 4 = oxygen saturation, 5 = level of consciousness measured on the ‘Alert’, ‘Verbal response’, ‘response to Pain’, and ‘Unresponsive’ (AVPU) scale, 6 = lower chest wall indrawing (signs and symptoms in the assessment domain), 7 = pneumonia severity classification (diagnosis domain), 8 = oral amoxicillin prescription to treat pneumonia, and 9 = oral amoxicillin dosage and frequency of administration (treatment domain). While these indicators were measured on different scales reflecting different aspects of care, we created a binary variable for each one of them as appropriate (Table 1). For each case record, we assessed documentation of cough and difficult breathing (primary pneumonia signs and symptoms required for identification of pneumonia cases), respiratory rate, oxygen saturation, AVPU, lower chest wall indrawing (secondary signs and symptoms required for classification of pneumonia severity).^26^ For each sign and symptom, we created a binary variable with the value one representing documentation in pediatric admission record (PAR) (e.g., cough assessed at point of admission and marked in a check box as present or absent) and zero representing lack of documentation of a sign and symptom in the medical record by the admitting clinician (Table 1). In the diagnosis domain, we assessed whether clinical pneumonia diagnosis and the severity classification documented in a patient’s PAR by the admitting clinician was in line with the diagnosis and the severity implied by presenting signs and symptoms. Here, we created a binary variable with value one representing correct diagnosis and severity classification and zero representing misclassification of pneumonia severity (Table 1).

In the treatment domain, we had two binary indicators, one assessing adherence to prescription guidelines and the other assessing adherence to dosing guidelines. For the prescription indicator, the value one represented prescription of oral amoxicillin to treat pediatric pneumonia as per the guidelines while zero represented deviation from ideal care (i.e., lack of evidence in a patient’s medical record that oral amoxicillin was prescribed) (Table 1).

To determine correctness of dose among oral amoxicillin recipients, we considered actual dose prescribed, patient’s weight and frequency of administration as documented in a patient’s medical record. We created a binary indicator with value one representing oral amoxicillin correct dosage and correct frequency of administration (i.e., 32–48 international units per Kilogram [IU/Kg] every 12 h). Inappropriate oral amoxicillin dosing was considered as: lack of documentation of actual oral amoxicillin dose prescribed, lack of documentation of patient’s weight, undocumented/wrong frequency of oral amoxicillin administration, oral amoxicillin underdosing (<32 IU/Kg every 12 h) or overdosing (>48 IU/Kg every 12 h) (Table 1).

### 3.2 Covariates

In this analysis, the covariates of intertest included intervention arm, follow-up time (in months) and their interaction, hospital malaria prevalence status and pediatric admission workload. Five out of 12 hospitals were drawn from high malaria endemic regions while the remaining seven hospitals were drawn from regions with low malaria endemicity in Kenya.^28^ Hospitals with less than 1000 pediatric admissions per year were categorized as low admission workload while those with 1000 or more pediatric admissions per year were categorized as high admission workload hospitals. This categorization allowed us to assess the impact of admission workload on quality of inpatient pediatric pneumonia care. This is considering that public hospitals in LMICs are often characterized by a shortage in workforce, potentially impeding delivery of health care services.^29–31^ At clinician level, gender and cadre were considered (here cadre refers to clinician’s level of training, that is, clinical officers with diploma-level training and medical officers with bachelors’ degree level training). Among 295 clinicians with observed cadre, majority were clinical officer interns at 62.4% (n = 184) followed by medical officer interns at 33.4% (n = 99). Clinical officer and medical officers accounted for 2.0% (n = 6) each. Among 296 clinicians with observed gender, 43.2% (n = 128) were females.

At patient level, we considered gender, number of comorbid illnesses and age in months at point of admission. While the WHO pediatric pneumonia treatment guidelines apply for children aged 2–59 months^26^ we categorized patients into two age groups, (i.e., 2–11 months and 12–59 months) in order to assess whether pneumonia care administered varied between infants and older children. This is considering that older children tend to have better outcomes compared to infants.^32^ Approximately, 42.5% (903/2127) of the patients were aged between two and 11 months and 57.5% (1224/2127) were females and among 2114 patients with observed gender, 55.1% (n = 1164) were males. Regarding comorbidities, we determined the total number of clinical diagnoses documented in patient medical records. The diagnoses of interest in the comorbidity variables included malaria, malnutrition, asthma, tuberculosis (TB), rickets, anemia, diarrhea and dehydration. For each patient, we created separate binary variables for each diagnosis above with value 1 denoting the presence of a disease and 0 denoting absence of a disease. We then created a categorical variable which consisted of a count of comorbidities defined as (0 = none, 1 = one, 2 = two, 3 = three or more comorbid illnesses). The above categorization was to assess whether care among pneumonia patients varied with an increase in the number of comorbid illness. Clinically, 46.8% (995/2127) of the patients had no comorbidities, 29.8% (633/2127) had one comorbidity, 17.9% (381/2127) had two comorbidities, and 5.5% (118/2127) had at least three comorbidities.

### 3.3 Missingness in the trial data

Missing data occurred in patient- and clinician level covariates. Approximately, 21.9% (83/378) and 21.7% (82/378) clinicians had missing data on the gender and cadre variables respectively, while patient’s gender was missing in 0.7% (17/2127) case records. An assessment of the missing data pattern revealed that nearly all clinicians with missing cadre had gender missing as well.

## 4 Application: Model Fitting and Inference

### 4.1 Multiple imputation

Before fitting the analyses models of interest, we imputed partially observed covariates assuming a missing at random (MAR) mechanism. MI was conducted within joint modeling (JM) framework where imputation values are drawn from a multivariate normal distribution in a single step.^23,33^ We used the latent normal approach to impute incomplete categorical variables of interest.^23^ Multiple imputation was implemented in the *jomo*^34^ and *mitml*^35^ packages in R (version 3.5.4) which allow imputation of categorical variables with more than two levels in the second and higher levels of the multilevel structure. For the *i^th^* (*i* = 1,…,2127) patient nested within the *j^th^* clinician (*j* = 1,…,378) in hospital *l* (*l* = 1,…,12), we defined a two-level JM imputation model corresponding to

(7) $$\begin{array}{c} {\text{Y}}_{\text{ijl}}^{\left(1\right)}={\text{X}}_{\text{ijl}}^{\left(1\right)}\beta^{\left(1\right)}+b_{jl}^{\left(1\right)}+e_{ijl}^{\left(1\right)}\\ {\text{Y}}_{jl}^{\left(2\right)}={\text{X}}_{\text{jl}}^{\left(2\right)}\beta^{\left(2\right)}+b_{jl}^{\left(2\right)}\\ e_{ijl}^{\left(1\right)}∼N\left(0,\sigma_{e}^{2}\right),\;and\;\left(b_{jl}^{\left(1\right)},b_{jl}^{\left(2\right)}\right)∼N\left(0,\Sigma_{\text{b}}\right), \end{array}$$

where ${\text{Y}}_{\text{ijl}}^{\left(1\right)}$ and ${\text{Y}}_{\text{jl}}^{\left(2\right)}$ are vectors of partially observed level 1 variables (patient’s sex) and level two variables (clinician’s sex and cadre) respectively. Predictor variables $\left({\text{X}}_{\text{ijl}}^{\left(1\right)}\right)$ for missing patient’s sex were fully observed variables (i.e., follow-up time, interacted with feedback arm, hospital admission workload and hospital malaria prevalence status, patient’s age and the number of comorbid illnesses). Predictor variables $\left({\text{X}}_{\text{jl}}^{\left(2\right)}\right)$ for missing clinician’s sex and cadre at the second level of the imputation model included follow-up time interacted with feedback arm, hospital admission workload and hospital malaria prevalence status. We also included all the nine binary response variables in both levels of the imputation model. A random intercept (*b_jl_*) was included to account for clustering at clinician level. Missing values were imputed 20 times with a burn-in of 500, and 500 updates between each imputed data set. Imputed values were assessed as appropriate while trace plots were used to assess convergence of the imputation model.^36^

### 4.2 Separate univariate analyses

First, we analyzed the nine outcomes separately under complete case analysis and after multiple imputation of missing covariates. For each outcome (*r* = 1,2,…,9), we fitted a generalized linear mixed model defined by.

(8) $$\begin{array}{ll} logit\left[P\left(Y_{rijl}=1\right)\right] & =\beta_{r0}+\beta_{r1}x_{\left(age\;group;rijl\right)}+\beta_{r2}x_{\left(patient\;sex;\;rijl\right)}+\beta_{r3}x_{\left(comorbidities=0;rijl\right)}+\beta_{r4}x_{\left(comorbidities=1;rijl\right)}\\ \; & \;+\beta_{r5}x_{\left(comorbidities=2;rijl\right)}+\beta_{r6}x\left({\;}_{clinician\;cadre;rjl}\right)+\beta_{r7}x_{\left(clinician\;sex;rjl\right)}+\beta_{r8}x_{\left(admission\;worklod;rjl\right)}\\ \; & \;+\beta_{r9}x_{\left(malaria\;prevalence;rl\right)}+\beta_{r10}x_{\left(time\;in\;months;rl\right)}+\beta_{r11}x_{\left(trial\;arm;rl\right)}+\beta_{r12}x_{\left(time\;in\;month;rl\right)}*x_{\left(trial\;arm;rl\right)}+b_{rijl},. \end{array}$$

where *β*_*r*1_,*β*_*r*2_…,*β*_*r*12_ are regression parameters associated with known fixed covariates for the *r^th^* outcome. Due to relatively low numbers of clinical and medical officers, we grouped clinicians into two cadres from the initial four. That is, clinical officers (CO) combine clinical officers and clinical officer interns and medical officers (MO) combine medical officers and medical officer interns, respectively. The vector of random clinicians’ intercepts *b_ijl_* is assumed to follow a normal distribution with mean zero and variance $\sigma_{b}^{2}$.

### 4.3 Full multivariate joint model

To analyze the nine pneumonia outcomes jointly, a full multivariate joint model was considered:

(9) $$\begin{array}{c} logit\left[P\left(Y_{1i}=1\right)\right]={\text{X}}_{i}\beta_{1}+b_{1i}\\ logit\left[P\left(Y_{2i}=1\right)\right]={\text{X}}_{i}\beta_{2}+b_{2i}\\ \vdots \\ logit\left[P\left(Y_{9i}=1\right)\right]={\text{X}}_{i}\beta_{9}+b_{9i}, \end{array}$$

where ***X***_*i*_ denotes a vector of known covariates and ***β***_1_, ***β***_2_,…,***β***_9_ are vectors of regression parameters to be estimated for each of the nine outcomes. The random clinicians’ intercepts were assumed to follow a joint zero-mean normal distribution denoted by

$$\left(\begin{array}{l} b_{1i}\\ b_{2i}\\ b_{3i}\\ b_{4i}\\ b_{5i}\\ b_{6i}\\ b_{7i}\\ b_{8i}\\ b_{9i} \end{array}\right)∼N\left(0,\text{D}\right)$$

where D, the covariance matrix of the random effects has the following structure:

(10) $$\text{D}=\left[\begin{array}{ccccccccc} \sigma_{b_{1}}^{2} & \sigma_{b_{1}b_{2}} & \sigma_{b_{1}b_{3}} & \sigma_{b_{1}b_{4}} & \sigma_{b_{1}b_{5}} & \sigma_{b_{1}b_{6}} & \sigma_{b_{1}b_{7}} & \sigma_{b_{1}b_{8}} & \sigma_{b_{1}b_{9}}\\ \; & \sigma_{b_{2}}^{2} & \sigma_{b_{2}b_{3}} & \sigma_{b_{2}b_{4}} & \sigma_{b_{2}b_{5}} & \sigma_{b_{2}b_{6}} & \sigma_{b_{2}b_{7}} & \sigma_{b_{2}b_{8}} & \sigma_{b_{2}b_{9}}\\ \; & \; & \sigma_{b_{3}}^{2} & \sigma_{b_{3}b_{4}} & \sigma_{b_{3}b_{5}} & \sigma_{b_{3}b_{6}} & \sigma_{b_{3}b_{7}} & \sigma_{b_{3}b_{8}} & \sigma_{b_{3}b_{9}}\\ \; & \; & \; & \sigma_{b_{4}}^{2} & \sigma_{b_{4}b_{5}} & \sigma_{b_{4}b_{6}} & \sigma_{b_{4}b_{7}} & \sigma_{b_{4}b_{8}} & \sigma_{b_{4}b_{9}}\\ \; & \; & \; & \; & \sigma_{b_{5}}^{2} & \sigma_{b_{5}b_{6}} & \sigma_{b_{5}b_{7}} & \sigma_{b_{5}b_{8}} & \sigma_{b_{5}b_{9}}\\ \; & \; & \; & \; & \; & \sigma_{b_{6}}^{2} & \sigma_{b_{6}b_{7}} & \sigma_{b_{6}b_{8}} & \sigma_{b_{6}b_{9}}\\ \; & \; & \; & \; & \; & \; & \sigma_{b_{7}}^{2} & \sigma_{b_{7}b_{8}} & \sigma_{b_{7}b_{9}}\\ \; & \; & \; & \; & \; & \; & \; & \sigma_{b_{8}}^{2} & \sigma_{b_{8}b_{9}}\\ \; & \; & \; & \; & \; & \; & \; & \; & \sigma_{b_{9}}^{2} \end{array}\right].$$

### 4.4 Pairwise joint modeling

To circumvent computational burden associated with model (9), we applied the pairwise approach to jointly model the probability of documentation among nine pneumonia outcomes under complete case analysis and after multiple imputation of missing covariates. We fitted 36 pairwise models where each pairwise model was defined by.

(11) $$\begin{array}{ll} logit\left[P\left(Y_{rijl}=1\right)\right] & =\beta_{r0}+\beta_{r1}x_{\left(age\;group;rijl\right)}+\beta_{r2}x_{\left(patient\;sex;rijl\right)}+\beta_{r3}x_{\left(comorbidities=0;rijl\right)}+\beta_{r4}x_{\left(comorbidities=1;rijl\right)}\\ \; & \;+\beta_{r5}x_{\left(comorbidities=2;rijl\right)}+\beta_{r6}x_{\left(clinician\;cadre;rjl\right)}+\beta_{r7}x_{\left(clinician\;sex;rjl\right)}+\beta_{r8}x_{\left(admission\;worklod;rjl\right)}\\ \; & \;+\beta_{r9}x_{\left(malaria\;prevalence;rl\right)}+\beta_{r10}x_{\left(time\;in\;months;rl\right)}+\beta_{r_{11}}x_{\left(trial\;arm;rl\right)}+\beta_{r12}x_{\left(time\;in\;months;rl\right)}*x_{\left(trial\;arm;rl\right)}+b_{rijl},. \end{array}$$

$$\begin{array}{ll} logit\left[P\left(Y_{sijl}=1\right)\right] & =\beta_{s0}+\beta_{s1}x_{\left(age\;group;sijl\right)}+\beta_{s2}x_{\left(patient\;sex;sijl\right)}+\beta_{s3}x_{\left(comorbidities=0;sijl\right)}+\beta_{s4}x_{\left(comorbidities=1;sijl\right)}\\ \; & \;+\beta_{s5}x_{\left(comorbidities=2;sijl\right)}+\beta_{s6}x_{\left(clinician\;cadre;sjl\right)}+\beta_{s7}x_{\left(clinician\;sex;sjl\right)}+\beta_{s8}x_{\left(admission\;worklod;sjl\right)}\\ \; & \;+\beta_{s9}x_{\left(malaria\;prevalence;sl\right)}+\beta_{s10}x_{\left(time\;in\;months;sl\right)}+\beta_{s_{11}}x_{\left(trial\;arm;sl\right)}+\beta_{s12}x_{\left(time\;in\;months;sl\right)}*x_{\left(trial\;arm;sl\right)}+b_{sijl}, \end{array}$$

where *Y_rijl_* and *Y_sijl_* denote the *r^th^* and the *s^th^* outcomes, ***r ≠ s*** for **(*r,s* = 1,2,…,9)** for patient *i* admitted by clinician *j* in hospital *l*. Each outcome occurred in eight specific pairs and we included a random clinicians’ intercept in each model. For each pairwise joint model, the random effects were assumed to follow a bivariate normal distribution denoted by

(12) $$\left(\begin{array}{c} b_{r}\\ b_{s} \end{array}\right)∼N\left[0,\left(\begin{array}{cc} \sigma_{b_{r}}^{2} & \sigma_{b_{r}b_{s}}\\ \; & \sigma_{b_{s}}^{2} \end{array}\right)\right].$$

We fitted all pairwise joint models using the *JMbayes* package^37^ using a server with the following specification: 40 GB memory, Intel Xeon E5-4650 (2.70GHz) processor (12 cores/24 threads), Gnu/Linux Ubuntu 14.04 OS, and R (version 3.4.4) programming language. For verification purposes, complete case analysis was also conducted in SAS version 9.4 using a SAS macro provided by.^10^

Under complete case analysis, regression estimates, and standard errors were averaged across 36 pairwise models using the pseudo-likelihood approach presented in Section 2. Likewise, regression parameters were averaged across the various pairwise models for each imputed data set. Variance–covariance estimators^6^ were also obtained for each imputed data set. This step resulted in 20 sets of averaged regression parameters and variance–covariance estimators respectively. Thereafter, Rubin’s rules^38^ were applied to obtain final estimates while accounting for within and between imputation variability. More details on the two-step procedure are as follows.

#### 4.4.1 Inference for fixed regression parameters

Each bivariate model in the *m^th^* imputed dataset had a vector of 26 regression coefficients (i.e., 13 regression coefficients for each outcome) denoted by ${\hat{\beta }}_{qm},q=1,2,\ldots ,36$, *m* = 1,2,…,20. We stacked the 36 pairwise parameter estimate vectors resulting into a column vector with 936 rows, that is,

$${\hat{\beta }}_{m}={\left[\begin{array}{c} {\hat{\beta }}_{1m}\\ {\hat{\beta }}_{2m}\\ \vdots \\ {\hat{\beta }}_{36m} \end{array}\right]}_{936\times 1},\;m=1,2,\ldots ,20.$$

Any two pairwise joint models with a common outcome (e.g., *l*(*Y_r_*, *Y_s_*) and *l*(*Y_r_*, *Y_s′_*) *s* ≠ *s′*) shared the parameters for the ***r**^th^* outcome.^8–10,24^ To account for duplicate parameter estimates, we pre-multiplied ${\hat{\beta }}_{\text{m}}$ with an appropriate weight matrix ***A*** as follows,

(13) $${\hat{\beta }}_{m}^{*}=\text{A}{\hat{\beta }}_{m},\;m=1,2,\ldots ,20.$$

The weight matrix ***A*** had 117 rows (i.e., 13 regression parameters for each of the nine outcomes) and 936 columns and it was constructed such that, it averaged all duplicate parameter estimates of an outcome across the eight pairwise models in which it occurred. The resulting vector, ${\hat{\beta }}_{m}^{*}$ was a stacked column vector of 117 parameter estimates for all nine outcomes. Each outcome had 13 regression parameters denoted by ${\hat{\beta }}_{mr}^{*}$. This step was repeated for all 20 imputed data sets.

#### 4.4.2 Inference for standard errors

The corresponding standard errors were obtained using the pseudo-likelihood approach introduced above. For each bivariate pair, *Y_mq_*, *q* = 1,2,…,36, in the *m^th^* imputed dataset, we estimated the variance–covariance matrix, ***H***^–1^***GH***^–1^. Since ***H*** and ***G*** depend on the unknown parameters in ***Θ***,^8,24^ estimation proceeded as follows. N indicates the total number of subjects.

**Step 1:** We obtained ${\hat{\text{J}}}_{mq}$ and ${\hat{\text{K}}}_{mq}$ for each pairwise model using.
  $${\hat{\text{J}}}_{mq}=\sum_{i=1}^{N}{\text{X}}_{imq}^{T}{\hat{\text{T}}}_{imq}{\text{X}}_{imq}\;\text{and}\;{\hat{\text{K}}}_{mq}=\left[{\text{X}}_{1mq}^{T}{\hat{\text{T}}}_{1mq},{\text{X}}_{2mq}^{T}{\hat{T}}_{2mq},\ldots ,{\text{X}}_{Nmq}^{T}{\hat{\text{T}}}_{Nmq}\right],$$
  where ***X***_*imq*_1_ corresponds to the *i^th^* subject’s contribution in the design matrix for the fixed effects, ${\hat{\text{T}}}_{imq}=\left(Z_{imq}{\hat{\text{D}}}_{mq}Z_{imq}^{T}\right)$ where *Z_imq_* is the *i^th^* subject’s contribution in the design matrix for random effects^24^ and ***D**_mq_* is the variance-covariance matrix for the random effects for the *q^th^* pair in the *m^th^* imputed data set. N indicates the number of subjects.
**Step2:** We combined ${\hat{\text{J}}}_{mq}$ and ${\hat{\text{K}}}_{mq}$ estimated across all the 36 pairs, (i.e., $\left({\hat{\text{J}}}_{m1},{\hat{\text{K}}}_{m1}\right),\left({\hat{\text{J}}}_{m2},{\hat{\text{K}}}_{m2}\right),\ldots ,\left({\hat{\text{J}}}_{m36},{\hat{\text{K}}}_{m36}\right)$) as follows.
  $${\hat{\text{J}}}_{m}={\left[\begin{array}{cccc} {\hat{\text{J}}}_{\text{m}1} & 0 & \cdots & 0\\ 0 & {\hat{\text{J}}}_{\text{m}2} & \cdots & 0\\ \vdots & \vdots & \ddots & \vdots \\ 0 & \cdots & \cdots & {\hat{\text{J}}}_{\text{m}36} \end{array}\right]}_{936\times 936}\;\text{and}\;{\hat{\text{K}}}_{m}={\left[\begin{array}{c} {\hat{\text{K}}}_{m1}\\ {\hat{\text{K}}}_{m2}\\ \vdots \\ {\hat{\text{K}}}_{m36} \end{array}\right]}_{936\times \text{N}.}$$
**Step 3:** We estimated *H_m_* and *G_m_* as follows.
  $${\hat{\text{H}}}_{m}=\frac{1}{N}{\hat{\text{J}}}_{m}\;\text{and}\;{\hat{\text{G}}}_{m}=\frac{1}{N}{\hat{\text{K}}}_{m}{\hat{\text{K}}}_{m}{\;}^{T},$$
  where *N* is defined above.
**Step 4:** We obtained a variance–covariance matrix, ${\hat{\Sigma }}_{m}^{*}$ for each imputed dataset using.
  (14) $${\hat{\Sigma }}_{m}^{*}=\text{A}{\hat{\Omega }}_{m}{\text{A}}^{T},\;m=1,2,\ldots ,20,.$$
  where ${\hat{\Omega }}_{m}={\hat{\text{H}}}_{m}^{-1}{\hat{\text{G}}}_{m}{\hat{\text{H}}}_{m}^{-1}$ and ***A*** is the weight matrix defined above. Each ${\hat{\Sigma }}_{m}^{*}$ was a 117 × 117 covariance matrix and the diagonal elements corresponded to variances of fixed regression parameters in ${\hat{\beta }}_{m}^{*}$.

#### 4.4.3 Pooling final estimates

In the final step, we pooled the final estimates using Rubin’s rules^38^ for each of the 9 outcomes. This was based on the set of pairwise regression parameters and the estimated variance covariance matrices ${\hat{\Sigma }}_{m}^{*}$ estimated in.^10^ The pooled MI estimator for ***β*** is given by

(15) $$\bar{\beta_{r}^{*}}=\frac{1}{M}\sum_{m=1}^{M}{\hat{\beta }}_{mr}^{*},$$

with variance estimator

$${\hat{V}}_{r}={\hat{W}}_{r}+\left(\frac{M+1}{M}\right)\times {\hat{B}}_{r},$$

where

$${\hat{W}}_{r}=\frac{1}{M}\sum_{m=1}^{M}{\hat{\sigma }}_{mr}^{2}$$

is the average imputation variance, ${\hat{\sigma }}_{mr}^{2}$ are the diagonal elements of ${\hat{\Sigma }}_{m}^{*}$ and

$${\hat{B}}_{r}=\frac{1}{M-1}\sum_{m=1}^{M}{\left({\hat{\beta }}_{mr}^{*}-{\bar{\beta }}_{r}^{*}\right)}^{2}$$

is the between imputation variance. Final MI estimates were compared to those obtained under complete case analysis.

#### 4.4.4 Wald test for joint covariates effects under complete case analysis and after multiple imputation

To test for the joint effects of covariates on the outcomes, we used a Wald-type test under complete case analysis and after multiple imputation of missing covariates. The general linear hypothesis corresponded to.

(16) $$H_{0}:\text{L}\beta =0\;\text{vs}\;H_{A}:\text{L}\beta \neq 0.$$

Systems of linear equations were defined as appropriate for different parameter vectors. For illustration, the joint null hypothesis for the interaction effect between the intervention arm and follow-up time on the nine outcomes (i.e., *β*_1,12_ = *β*_2,12_ = *β*_3,12_ = *β*_4,12_ = *β*_5,12_ = *β*_6,12_ = *β*_7,12_ = *β*_8,12_ = *β*_9,12_ = 0) was defined using a system of linear equation below:

$${\left(\begin{array}{ccccc} 0 & 0 & 0 & 0 & 0\\ 0 & 0 & 0 & 0 & 0\\ \vdots & \vdots & \vdots & \vdots & \vdots \\ 0 & 0 & 0 & 0 & 0 \end{array}\;\begin{array}{ccccc} 0 & 0 & 0 & 0 & 0\\ 0 & 0 & 0 & 0 & 0\\ \vdots & \vdots & \vdots & \vdots & \vdots \\ 0 & 0 & 0 & 0 & 0 \end{array}\;\begin{array}{ccccc} 0 & 0 & 1 & 0 & 0\\ 0 & 0 & 0 & 0 & 0\\ \vdots & \vdots & \vdots & \vdots & \vdots \\ 0 & 0 & 0 & 0 & 0 \end{array}\;\begin{array}{ccccc} 0 & 0 & 0 & 0 & 0\\ 0 & 0 & 0 & 0 & 0\\ \vdots & \vdots & \vdots & \vdots & \vdots \\ 0 & 0 & 0 & 0 & 0 \end{array}\;\begin{array}{ccccc} 0 & 0 & 0 & 0 & 0\\ 0 & 0 & 0 & 0 & 0\\ \vdots & \vdots & \vdots & \vdots & \vdots \\ 0 & 0 & 0 & 0 & 0 \end{array}\;\begin{array}{ccccc} 0\ldots & 0 & 0 & 0 & 0\\ 1\ldots & 0 & 0 & 0 & 0\\ \ldots & \vdots & \vdots & \vdots & \vdots \\ 0\ldots & 0 & 0 & 0 & 0 \end{array}\;\begin{array}{ccccc} 0 & 0 & 0 & 0 & 0\\ 0 & 0 & 0 & 0 & 0\\ \vdots & \vdots & \vdots & \vdots & \vdots \\ 0 & 0 & 0 & 0 & 0 \end{array}\;\begin{array}{cccc} 0 & 0 & 0 & 0\\ 0 & 0 & 0 & 0\\ \vdots & \vdots & \vdots & \vdots \\ 0 & 0 & 0 & 1 \end{array}\right)}_{9\times 117}{\left(\begin{array}{c} \beta_{1,0}\\ \beta_{1,1}\\ \vdots \\ \beta_{1,12}\\ \beta_{2,0}\\ \beta_{2,1}\\ \vdots \\ \beta_{2,12}\\ \vdots \\ \vdots \\ \beta_{9,0}\\ \beta_{9,1}\\ \vdots \\ \beta_{9,12} \end{array}\right)}_{117\times 1}={\left(\begin{array}{c} 0\\ 0\\ \vdots \\ 0 \end{array}\right)}_{9\times 1}.$$

The alternative hypothesis stated that at least one of the parameters differs from zero. Under complete case analysis, the test statistic for the joint interaction term was calculated using

(17) $$F=\frac{1}{9}{\left({\text{L}}_{12}{\hat{\beta }}^{*}-0\right)}^{\prime }{\left({\text{L}}_{12}{\hat{\Sigma }}^{*}{{\text{L}}^{\prime }}_{12}\right)}^{-1}\left({\text{L}}_{12}{\hat{\beta }}^{*}-0\right),$$

where **L**_12_ is a matrix of zeros and ones defined to eliminate all parameter estimates except those associated with the interaction term (*i.e.*,***β***_*r*,12_ (*r* = 1,2,…,9)), ${\hat{\beta }}^{*}$ is a stacked vector of parameter estimates averaged across 36 pairwise models and ${\hat{\Sigma }}^{*}$ is the variance–covariance matrix estimated using pseudo-likelihood. Wald-test statistics for the other variables were calculated in a similar manner but adjusting the L matrix appropriately.

For imputed datasets, the joint null hypotheses were tested using linear systems of equations like those defined under complete case analysis. Nonetheless, the test statistics were calculated differently. For instance, the test statistic for the joint interaction term effect on the nine outcomes after multiple imputation was calculated using

(18) $$F=\frac{1}{9}{\left({\text{L}}_{12}{\bar{\beta }}_{m}^{*}-0\right)}^{\prime }{\left({\text{L}}_{12}{\hat{\text{V}}}_{m}{{\text{L}}^{\prime }}_{12}\right)}^{-1}\left({\text{L}}_{12}{\bar{\beta }}_{m}^{*}-0\right),$$

where ${\bar{\beta }}_{m}^{*}$ is a stacked vector of pooled parameter estimates for all the nine outcomes and ${\hat{\text{V}}}_{m}$ is the variance–covariance matrix based on Rubin’s rules. Wald-test statistics for the other variables were calculated in a similar manner but adjusting the L matrix appropriately. In each case, the test statistic was multiplied by nine (removing the fraction in front) resulting in test statistics that were distributed according to chi-squared distribution with nine degrees of freedom. A 5% level of significance was considered in all statistical tests.

#### 4.4.5 Association among pneumonia outcomes

The strength of association among documentation of pneumonia care indicators was evaluated using the variance–covariance matrix of the random-effects. Since the covariance matrix ***D*** defined in (10) was not estimated directly at analysis stage, we constructed it using blocks of random-effects variance–covariance matrices in (12) estimated in the pairwise joint models. Under multiple imputation, we first averaged duplicate variance across 36 pairwise random intercept models for each of the 20 imputed data set. Specifically, we extracted the random intercepts variance–covariance matrix for all 36 pairwise joint models, that is,

$${\text{D}}_{m1}=\left[\begin{array}{cc} \sigma_{b_{m1}}^{2} & \sigma_{b_{m1}b_{m2}}\\ \; & \sigma_{b_{m2}}^{2} \end{array}\right],\;{\text{D}}_{m2}=\left[\begin{array}{cc} \sigma_{b_{m1}}^{2} & \sigma_{b_{m1}b_{m3}}\\ \; & \sigma_{b_{m3}}^{2} \end{array}\right],\ldots ,{\text{D}}_{m36}=\left[\begin{array}{cc} \sigma_{b_{m8}}^{2} & \sigma_{b_{m8}b_{m9}}\\ \; & \sigma_{bm9}^{2} \end{array}\right].$$

We then created an overall variance-covariance matrix ***D_m_*** for each imputed data set accounting for overlapping information. For example, in each imputed data set, (***m*** = 1,2,…,20), documentation of cough occurred in the variance-covariance matrices of the first eight pairs, that is,

$${\text{D}}_{m1}=\left[\begin{array}{cc} \sigma_{b_{m1}}^{2} & \sigma_{b_{m1}b_{m2}}\\ \; & \sigma_{b_{m2}}^{2} \end{array}\right],\;{\text{D}}_{m2}=\left[\begin{array}{cc} \sigma_{b_{m1}}^{2} & \sigma_{b_{m1}b_{m3}}\\ \; & \sigma_{b_{m3}}^{2} \end{array}\right],\ldots ,{\text{D}}_{m8}=\left[\begin{array}{cc} \sigma_{b_{m1}}^{2} & \sigma_{b_{m1}b_{m9}}\\ \; & \sigma_{bm9}^{2} \end{array}\right].$$

We extracted the random intercept variances of each outcome from the pairs it occurred in and averaged them into a single random intercept variance estimate of *Y_r_* (e.g., $\sigma_{b_{1}}^{2}$ denoting the random intercept variance for cough). On the other hand, unique off-diagonal elements corresponding to covariance between any two outcomes were also mapped into ***D_m_***. Thereafter, we averaged all the 20 ***D_m_*** matrices, *m* = 1,2,…,20 to obtain the overall 9 × 9 variance covariance matrix ***D*** for all the nine outcomes. We used the same procedure to construct the random-intercepts variance–covariance under complete case analysis where we averaged duplicate variances across 36 pairwise random intercept models. The strength of association between any 2 outcomes, say *Y_r_* and *Y_s_* was calculated using

(19) $$\text{corr}\left(b_{r},b_{s}\right)=\frac{Cov\left(b_{r},b_{s}\right)}{\sqrt{Var\left(b_{r}\right)\times Var\left(b_{s}\right)}}=\frac{\sigma_{b_{r}b_{s}}}{\sqrt{\sigma_{b_{r}}^{2}\times \sigma_{b_{s}}^{2}}}.$$

We performed principal component analysis (PCA) on random clinicians’ intercepts variance–covariance matrices obtained under complete case analysis and after multiple imputation. This was to help visualize factor loadings among pneumonia outcomes of interest and how they correlated with one another.

## 5 Results

The level of documentation and adherence to recommended pneumonia care varied within and across domains of care. To be specific, most of the signs and symptoms in the assessment domain were well documented except for oxygen saturation and respiratory rate which had documentation rates of 60.9% (1297/2127) and 88.8% (1889/2127) respectively. On the other hand, the level of documentation and adherence to recommended guidelines in diagnosis and treatment domains, respectively was poor compared to that of signs and symptoms in the assessment domain. Specifically, of all 2127 syndromic pneumonia cases, only 1473 (69.3%) had correct clinical pneumonia diagnosis and severity classification documented in the medical record. In the treatment domain, about 48.7% (1036/2127) were prescribed with oral amoxicillin as per the guidelines. However, only 25% (523/2127) of all pneumonia patients got the right oral amoxicillin dose and in the right frequency of administration, that is, 32–48 international units/Kilogram (IU/Kg) every 12 h.

## 6 Wald-Type Tests for Joint Covariates Effects

After multiple imputation of missing clinician- and patient level covariates, the Wald-type test suggested a significant joint interaction effect between the trial arm and follow-up time on documentation and adherence to recommended clinical guidelines on all the nine pediatric pneumonia outcomes of interest (P-value <0.05). Likewise, pediatric admission workload and malaria prevalence status at hospital level also exhibited significant joint effects on all the nine outcomes (Table 2). At clinician level, gender and cadre had significant joint effect on documentation and adherence to recommended pediatric pneumonia care guidelines (Table 2). At patient level, age and comorbidity had significant joint effect on documentation of all the nine outcomes. On the other hand, patient’s gender did not have a significant joint effect on the outcomes of interest (Table 2). The Wald-type test results under complete case analysis were consistent with those from imputed datasets for all the covariates. That is, all the covariates had significant joint effects on the nine outcomes except patient’s gender (Table 2).

**Figure 1:**
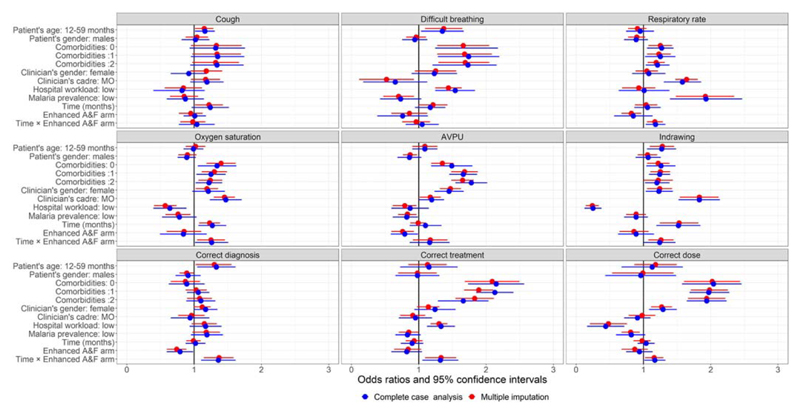
Odds ratios (dots) and 95% confidence intervals (horizontal bars) under complete case analysis and after multiple imputation of missing covariates: Pairwise joint modeling of nine pneumonia care outcomes

**Figure 2:**
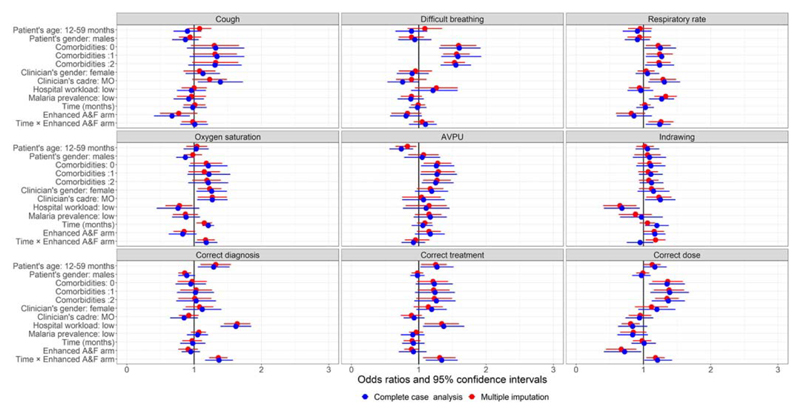
Odds ratios (dots) and 95% confidence intervals (horizontal bars) under complete case analysis and after multiple imputation of missing covariates: Separate univariate analysis of nine pneumonia care outcomes

Figure 1 and Supplementary Table A1-A2 present the odds ratios and their 95% confidence intervals estimated from the pairwise joint model under complete case analysis and after multiple imputation of missing covariates. Separate univariate analyses results are presented in Figure 2 and Supplementary Table A3-A4.

Under pairwise joint modeling, the magnitude and direction of covariates effects varied among pneumonia outcomes of interest. Over time, documentation and adherence to recommended clinical guidelines improved in six out of nine pneumonia care indicators among children admitted to six intervention hospitals (i.e., enhanced audit and feedback arm). That is, for a unit increase in follow-up month, the change in the adjusted odds of oxygen saturation, respiratory rate, lower wall indrawing documentation (in the assessment domain), correct pneumonia diagnosis, oral amoxicillin prescription and correct dosage among patients admitted to intervention hospitals (i.e., enhanced A&F arm) were significantly more positive in comparison to the change among patients admitted to control hospitals. These observations were made under complete case analysis and after multiple imputation of missing clinician- and patient level covariates (Figure 1). Nevertheless, the estimated 95% confidence intervals estimated were consistently narrower after multiple imputation. On the other hand, there was no significant difference in the documentation of cough, difficult breathing and AVPU over time between patients admitted to six intervention hospitals (enhanced A&F arm) and patients admitted to six control hospitals (standard A&F arm).

We also observed a few instances of contrasting results. For example, after multiple imputation, the adjusted odds of AVPU documentation were significantly lower among patients admitted to hospitals with low pediatric admission workload (Figure 1 and supplementary Table A1). Under complete case analysis, however, there was no significant difference (Figure 1, supplementary Table A2). Similarly, the adjusted odds of documentation of difficult breathing and correct oral amoxicillin dose among patients admitted in low malaria prevalence hospitals were lower compared to the odds of patients admitted in high malaria hospitals. However, under complete case analysis, the difference was not statistically significant (Figure 1, supplementary Table A2).

With regards to separate univariate analysis, the direction and magnitude of effects of most of the covariates across the nine outcomes were by and large consistent with those observed under a pairwise joint model. Additionally, it was found that documentation and adherence to recommended clinical guidelines improved over time in five out of nine pneumonia care indicators among children admitted to six hospitals in the enhanced A&F arm (intervention arm). To be specific, for a unit increase in follow-up month, the change in the adjusted odds of oxygen saturation, respiratory rate, correct pneumonia diagnosis, oral amoxicillin prescription and correct dosage among patients admitted to intervention hospitals were significantly more positive in comparison to the change among patients admitted to control hospitals. These observations were made under complete case analysis and after multiple imputation. However, multiple imputation improved precision of the estimated odds ratios compared to complete case analysis (Figure 2, Supplementary Tables A3-A4). The estimated variance among admitting clinicians (i.e., variance between random clinicians’ intercepts) varied across the nine pneumonia outcomes, both under complete case analysis and after multiple imputation of missing covariates (Tables A3, A4).

Tables 3 and 4 present variance-correlation matrices of random clinicians’ intercepts among 9 pneumonia outcomes under complete case analysis and after multiple imputation, respectively. Generally, the magnitude of correlation estimated among outcomes was consistently larger under multiple imputation compared to complete case analysis. Moreover, the strength and direction of association among outcomes varied within and across domains of care. For instance, the strength of association between documentation of oxygen saturation and respiratory rate was somewhat high, compared to association with other indicators in the assessment domain. To be specific, correlation between oxygen saturation and respiratory rate documentation increased from 0.69 (Table 3) under complete case analysis to 0.89 (Table 4) after multiple imputation of missing covariates. In the treatment domain, prescription of oral amoxicillin and correct dosage exhibited a strong positive association with a correlation coefficient of 0.73 under complete case analysis (Table 3) and 0.80 after multiple imputation of missing covariates (Table 4).

Across domains of care, correct pneumonia diagnosis was strongly associated with prescription of oral amoxicillin and correct dosage both in the treatment domain. We also observed that documentation of oxygen saturation, respiratory rate, and lower wall chest wall indrawing, in the assessment domain were positively associated with correct pneumonia diagnosis, amoxicillin prescription and correctness of the dose. These observations were made under complete case analysis (Table 3) and after multiple imputation (Table 4). On the other hand, documentation of cough and difficult breathing (primary pneumonia signs and symptoms) and AVPU in the assessment domain were negatively associated with documentation of other pneumonia care indicators.

**Figure 3:**
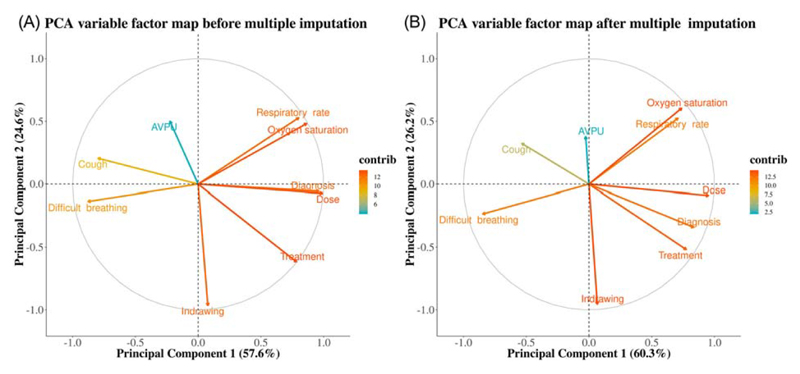
Results (component loadings for the first and second principal components) of a principal components analysis on correlation matrix of the random intercepts of model under complete case analysis (panel a) and after multiple imputation (panel b)

Under complete case analysis, a principal component analysis (PCA) on the correlation matrix of the random intercepts showed that the first and second principal components explained 57.6% and 24.6% of the variation respectively (Figure 3, panel a). After multiple imputation, the first and second principal components explained 60.3% and 26.2% of the variation respectively (Figure 3, panel b). Vectors of two positively correlated outcomes in the loading plots were close, forming a small angle between them (e.g., oxygen saturation and respiratory rate). On the other hand, vectors of negatively correlated outcomes (e.g., cough and treatment) were diverging forming a large angle between them. The direction of vectors for all the outcomes was consistent under complete case analysis and after multiple imputation.

## 7 Discussion

In this study we sought to estimate the joint and separate effects of covariates on nine pediatric pneumonia outcomes from a routine data set collected during a cluster randomized trial conducted in Kenyan hospitals. We also estimated the strength of association among the outcomes using a correlated random-effects joint model.^8,14^ Missing data in covariate across two level of hierarchy were handled using multiple imputation.

During the trial period, documentation and adherence to recommended pediatric pneumonia guidelines by clinicians depended on individual quality of care indicators. For instance, documentation of pneumonia care indicators, that did not require a lot of cognitive effort, were highly documented (e.g., cough, difficult breathing) compared to indicators that required more cognitive effort on the part of the clinician (e.g., prescribing the right treatment in the right dosage). These variations in delivery of quality care could also be due to hospital level factors, such as lack of or broken medical devices, impeding delivery of recommended care (e.g., pulse oximeter to measure oxygen saturation).

From Wald type test, we observed significant joint effects of all covariates of interest except patient’s gender and these observations were consistent between complete case analysis and after multiple imputation of missing patient and clinician level covariates. After fitting pairwise joint model, results showed that documentation and adherence to recommended clinical guidelines improved over time in six out of nine pneumonia care indicators among children admitted to six hospitals in the intervention arm. In separate analysis, documentation and adherence to recommended clinical guidelines improved over time in five out of nine pneumonia care indicators among children admitted to six hospitals in the intervention arm.

In both analysis approaches (i.e., pairwise joint modeling and separate univariate analysis), multiple imputation led to more precise estimates compared to those from complete case analysis. These observations were attributed to loss of information under complete case analysis resulting in larger standard errors hence wider 95% confidence intervals.

Further results revealed that the strength and direction of association among pneumonia outcomes varied within and across domains of care. Thus, an assumption of common random-effects among all outcomes would be too restrictive and unrealistic for pneumonia trial data analyzed in this study.

In the pairwise modeling approach, estimates obtained by averaging over several auxiliary estimates (from the various pairs) do not maximize the full multivariate likelihood. However, Fieuws and Verbeke^39^ demonstrated with simulations that the loss of efficiency is small in the pairwise approach relative to a full maximum-likelihood based approach. Moreover, the averaged estimates are consistent and asymptotically normal,^8^ a property which holds for imputed data sets thus, ensuring valid within imputation estimates. Validity of within imputation estimates is a prerequisite for the application of Rubin’s rules which then account for between imputation variability.^38^

Although we did not evaluate computational complexity explicitly, combining pairwise joint model fitting and multiple imputation comes with its computational expense as demonstrated in this study. At imputation stage, the level of complexity is compounded when missing data occur in more than one level of clustering. In such occurrences, it is paramount to account for the hierarchical structure present in the analysis model of interest in the imputation model as well. This is because incompatibility between imputation and analysis model may lead to biased estimates, underestimated cluster level variances and overestimated individual level variances.^23,33^ In the current study, missing covariates were imputed using the latent normal approach within the joint model imputation framework while accounting for clustering at clinician level. Additionally, the outcomes of interest, all fully observed were included in the imputation model as auxiliary variables. Nonetheless, there is need for further research possibly through a simulation study to evaluate compatibility between imputation and substantive model or the lack thereof, in the high dimensional joint modeling context.

At analysis stage, complexity stems from calculating parameters of interest (e.g., obtaining variance–covariance matrices for each imputed data set using the pseudolikelihood approach before applying Rubin’s rules). Besides, constructing the overall variance covariance matrix for the random effects is not straight forward, hence the need for greater care to avoid incorrect inferences due to miscalculations. Therefore, future studies can consider developing and incorporating generic functions and packages into standard statistical software to handle missing data and other computational aspects (e.g., Wald-type tests to test for joint covariate effects after multiple imputation) more efficiently when the substantive model of interest entails joint modeling of clustered and high-dimensional vectors of outcomes.

The correlated random-effects joint model fitted using the pairwise approach has been previously used to jointly analyze clustered binary data^10^ as well as continuous longitudinal outcomes.^14^ However, there is essentially no example in the literature on how to account for missing covariates in a high-dimensional joint modeling context. Additionally, we extended and exemplified Wald-type tests for joint covariate effects after multiple imputation in a high-dimensional joint modeling context. To our knowledge, there are no examples in the literature demonstrating application of Wald-type tests for joint covariate effects tests in high-dimensional joint modeling after multiple imputation, hence the novelty of this study.

Besides estimating the joint effects of covariates after multiple imputation, we estimated the strength of association among quality-of-care outcomes, aspects that are largely ignored in routine pediatric care studies. In previous analysis of the trial data, for instance, diagnosis and classification of pneumonia cases was the primary outcome of interest.^2^ In yet another study, pneumonia quality of care indicators were combined into a single ordinal composite outcome known as the pediatric quality of care indicator (PAQC) score.^5^ Therefore, when there is need for joint inference, we recommend this study as a practical example for handling high-dimensional vector of outcomes using a pairwise fitting approach and at the same time performing multiple imputation to account for missing covariates. However, if the research question does not necessitate joint inference, then univariate mixed models as tools for analysis suffice.^14^

Evidently, this study has several limitations. Firstly, we imputed missing covariates assuming a missing at random (MAR) mechanism, an assumption that cannot be verified using the observed data alone.^8,23,40^ Therefore, sensitivity analysis is recommended to explore the robustness of the inferences to the MAR assumptions.

As already noted, fitting pairwise joint models on multiply imputed data sets was time intensive. Future studies may consider multiple outputation, an approach suggested by^18^ as alternative to the pairwise joint modeling using a sandwich-type robust variance estimator.

In conclusion, there were significant joint effects of covariates on nine pneumonia outcomes before and after multiple imputation of missing covariates. In both pairwise joint modeling and separate univariate analysis approaches, enhanced audit and feedback improved documentation and adherence to recommended clinical guidelines over time in six and five out of nine pneumonia care outcomes of interest. Irrespective of the analysis approach, multiple imputation of missing covariates improved precision of parameter estimates compared to complete case analysis. The strength and direction of association estimated using clinicians’ random intercepts estimated from the pairwise joint model varied among pneumonia outcomes within and across the three domains of pneumonia care. Across domains of care, pneumonia diagnosis was strongly correlated with oral amoxicillin prescription and dosage.

## Supplementary Material

Appendix S1: Suplement Appendix Tables

## Figures and Tables

**Table 1:** Definition of binary outcomes in the assessment, diagnosis and classification and treatment domains of pediatric pneumonia care

Quality of care domain	Indicator	Scores in binary indicators	
1. Assessment	Cough	1:	if cough is documented,
*Primary signs and symptoms*			
		0:	if it is not documented.
	Difficult breathing	1:	if difficult breathing is documented,
*Secondary sign and symptoms*	Respiratory rate	0: 1:	if it is not documented. if respiratory rate is documented,
		0:	if it is not documented.
	Oxygen saturation	1:	if oxygen saturation is documented,
		0:	if it is not documented.
	AVPU^a^	1:	if AVPU is documented,
		0:	if it is not documented.
	Lower chest wall indrawing	1:	if indrawing is documented,
		0:	if it is not documented.
2. Diagnosis and classification	Correct diagnosis*	1: if the admitting clinician documented pneumonia as the clinical diagnosis 0: if documented clinical diagnosis is severe pneumonia or missing classification.	
3. Treatment	Correct prescription	1: if oral amoxicillin was prescribed and documented in the medical record. 0: if amoxicillin was not prescribed	
	Correct oral amoxicillin dose	1: if oral amoxicillin was prescribed in correct dose and frequencies, that is, 32-48 international units/Kilogram (IU/Kg) every 12 h. 0: if oral amoxicillin prescription is an under dose (<32 IU/Kg) or overdose (>48 IU/Kg) or missing amoxicillin dose or wrong frequency or missing frequency or missing patient’s weight.	

*Note*: AVPU^a^:-Alert, Verbal response, Pain response, Unresponsive

* Pneumonia diagnosis for patients with history of cough and/or difficult breathing (primary signs) in combination with signs of lower chest wall indrawing and/or respiratory rate (RR) ≥50 (≥40) for patients aged 2-11 (12-59 months), in the absence of danger any sign (inability to drink/breastfeed, cyanosis, grunting or oxygen saturation < 90% or AVPU = ‘V’, ‘P’ or ‘U’).

**Table 2:** Wald-type test results for joint effects of covariates on nine pneumonia outcomes

	Wald-type test under complete case analysis		Wald-type test after multiple imputation	
Effect	Test statistic	*p* value	Test statistic	*p* value
Patient’s age	19.62	0.02	21.81	0.01
Patient’s gender	12.20	0.21	13.16	0.15
Comorbidity	20.54	0.01	23.48	0.01
Clinician’s gender	20.91	0.01	22.47	0.007
Clinician’s cadre	19.94	0.02	17.96	0.03
Admission workload	25.56	0.002	24.73	0.003
Malaria prevalence	17.89	0.04	19.01	0.02
Time in months	19.26	0.02	18.16	0.03
Enhanced A&F^a^ arm	17.98	0.04	16.76	0.04
Enhanced A&F arm x follow-up time	18.13	0.03	23.11	0.005

*Note*: A&F^a^, Audit and feedback.

**Table 3:** Variance-correlation matrix for random clinicians' intercepts under complete case analysis

	Cough	Difficult breathing	Respiratory rate	Oxygen saturation	AVPU^a^	Indrawing	Correct diagnosis	Correct treatment	Correct dose
Cough	1.49								
Difficult breathing	0.07	1.92							
Respiratory rate	−0.29	−0.43	2.71						
Oxygen saturation	−0.17	−0.47	0.63	7.38					
AVPU	−0.14	−0.19	−0.20	0.09	2.26				
Indrawing	−0.22	−0.11	−0.54	−0.39	−0.19	2.33			
Correct diagnosis	−0.49	−0.53	0.48	0.29	−0.06	0.04	2.64		
Correct treatment	− 0.48	−0.42	0.07	0.16	−0.38	0.66	0.64	1.81	
Correct dose	−0.54	−0.64	0.57	0.69	−0.21	0.19	0.62	0.73	1.30

*Note*: AVPU^a^: Alert, verbal response, pain response, unresponsive.

**Table 4:** Variance-correlation matrix for random clinicians' intercepts after multiple imputation

	Cough	Difficult breathing	Respiratory rate	Oxygen saturation	AVPU^a^	Indrawing	Correct diagnosis	Correct treatment	Correct dose
Cough	1.05								
Difficult breathing	0.17	0.71							
Respiratory rate	−0.29	−0.60	2.47						
Oxygen saturation	−0.30	−0.78	0.89	2.23					
AVPU	−0.12	−0.24	−0.12	0.22	1.76				
Indrawing	−0.30	0.06	−0.52	−0.50	−0.26	1.82			
Correct diagnosis	−0.54	−0.65	0.40	0.24	−0.07	0.35	2.14		
Correct treatment	− 0.45	−0.55	0.23	0.26	−0.22	0.52	0.77	0.56	
Correct dose	− 0.47	−0.76	0.63	0.64	−0.18	0.15	0.74	0.80	0.67

*Note*: AVPU^a^, Alert, verbal response, pain response, unresponsive.

## Data Availability

The dataset analysed in this study are not publicly available because they are a property of the Ministry of Health and we do not have authority to share it on their behalf.

## References

[1] Gachau S, Ayieko P, Gathara D (2017). Does audit and feedback improve the adoption of recommended practices? Evidence from a longitudinal observational study of an emerging clinical network in Kenya. BMJ Glob Health.

[2] Ayieko P, Irimu G, Ogero M (2019). Effect of enhancing audit and feedback on uptake of childhood pneumonia treatment policy in hospitals that are part of a clinical network: a cluster randomized trial. Implementation Sci.

[3] Ogero M, Ayieko P, Boniface Makone TJ (2018). An observational study of monitoring of vital signs in children admitted to Kenyan hospi-tals: an insight into the quality of nursing care?. J Glob Health.

[4] Opondo C, Allen E, Todd J, English M (2016). The Paediatric admission quality of care (PAQC) score: designing a tool to measure the quality of early inpatient paediatric care in a low-income setting. Trop Med Int Health.

[5] Gachau S, Owuor N, Njagi EN, Ayieko P, English M (2019). Analysis of hierarchical routine data with covariate missingness: effects of Audit & Feedback on clinicians’ prescribed paediatric pneumonia care in Kenyan hospitals. Front Public Health.

[6] Ogero M, Akech S, Malla L, Agweyu A, Irimu G, English M (2020). Examining which clinicians provide admission hospital care in a high mortality setting and their adherence to guidelines: an observational study in 13 hospitals. Arch Dis Child.

[7] Profit J, Kowalkowski MA, Zupancic JA (2014). Baby-MONITOR: a composite indicator of NICU quality. Pediatrics.

[8] Molenberghs G, Verbeke G (2005). Models for Discrete Longitudinal Data.

[9] Fieuws S, Verbeke G, Molenberghs G (2007). Random-effects models for multivariate repeated measures. Stat Methods Med Res.

[10] Fieuws S, Verbeke G, Boen F, Delecluse C (2006). High dimensional multivariate mixed models for binary questionnaire data. J R Stat Soc Ser CAppl Stat.

[11] Verbeke G, Fieuws S, Molenberghs G, Davidian M (2014). The analysis of multivariate longitudinal data: a review. Stat Methods Med Res.

[12] Fitzmaurice G, Davidian M, Verbeke G, Molenberghs G (2008). Longitudinal data analysis.

[13] Fieuws S, Verbeke G (2004). Joint modelling of multivariate longitudinal profiles: pitfalls of the random-effects approach. Stat Med.

[14] Fieuws S, Verbeke G (2006). Pairwise fitting of mixed models for the joint modeling of multivariate longitudinal profiles. Biometrics.

[15] Gueorguieva R (2001). A multivariate generalized linear mixed model for joint modelling of clustered outcomes in the exponential family. Stat Model.

[16] McCulloch C (2008). Joint modelling of mixed outcome types using latent variables. Stat Methods Med Res.

[17] Faes C, Aerts M, Molenberghs G, Geys H, Teuns G, Bijnens L (2008). A high-dimensional joint model for longitudinal outcomes of different nature. Stat Med.

[18] Nassiri V, Ivanova A, Molenberghs G, Verbeke G (2017). Fast precision estimation in high-dimensional multivariate joint models. Biom J.

[19] Catalano PJ (1997). Bivariate modelling of clustered continuous and ordered categorical outcomes. Stat Med.

[20] Jaffa MA, Gebregziabher M, Jaffa AA (2014). A joint modeling approach for right censored high dimensional multivariate longitudinal data. J Biometric Biostat.

[21] Hickey GL, Philipson P, Jorgensen A, Kolamunnage-Dona R (2016). Joint modelling of time-to-event and multivariate longitudinal outcomes: recent developments and issues. BMC Med Res Methodol.

[22] Long JD, Mills JA (2018). Joint modeling of multivariate longitudinal data and survival data in several observational studies of Huntington’s disease. BMC Med Res Methodol.

[23] Carpenter JR, Kenward MG (2013). Multiple Imputation and its Applications.

[24] Kundu MG (2011). Implementation of pairwise fitting technique for analyzing multivariate longitudinal data in Sas.

[25] Ayieko P, Irimu G, English M (2017). Effect of enhanced feedback to hospitals that are part of an emerging clinical information network on uptake of revised childhood pneumonia treatment policy: study protocol for a cluster randomized trial. Trials.

[26] Organization WH (2013). Pocket Book of Hospital Care for Children: Guidelines for the Management of Common Childhood Illnesses.

[27] Harris PA, Taylor R, Thielke R, Payne J, Gonzalez N, Conde JG (2009). Research electronic data capture (REDCap)—a metadata-driven methodology and workflow process for providing translational research informatics support. J Biomed Inform.

[28] Ayieko P, Ogero M, Makone B (2015). Characteristics of admissions and variations in the use of basic investigations, treatments and outcomes in Kenyan hospitals within a new clinical information network. Arch Disease Childhood.

[29] Miseda MH, Were SO, Murianki CA, Mutuku MP, Mutwiwa SN (2017). The implication of the shortage of health workforce specialist on universal health coverage in Kenya. Hum Resour Health.

[30] English M, Strachan B, Esamai F (2020). The paediatrician workforce and its role in addressing neonatal, child and adolescent healthcare in Kenya. Arch Dis Child.

[31] Arsenault C, English M, Gathara D, Malata A, Mandala W, Kruk ME (2020). Variation in competent and respectful delivery care in Kenya and Malawi: a retrospective analysis of national facility surveys. Trop Med Int Health.

[32] Wang H, Liddell CA, Coates MM (2014). Global, regional, and national levels of neonatal, infant, and under-5 mortality during 1990–2013: a systematic analysis for the global burden of disease study 2013. The Lancet.

[33] Grund S, Lüdtke O, Robitzsch A (2018). Multiple imputation of missing data for multilevel models: simulations and recommendations. Org Res Method.

[34] Quartagno M, Grund S, Carpenter J (2019). Jomo: a flexible package for two-level joint modelling multiple imputation. R J.

[35] Grund S, Robitzsch A, Luedtke O, Grund MS (2019). Package ‘mitml’.

[36] Gelman A, Rubin DB (1992). Inference from iterative simulation using multiple sequences. Stat Sci.

[37] Rizopoulos D (2016). The R package JMbayes for fitting joint models for longitudinal and time-to-event data using MCMC. J Stat Software.

[38] Rubin DB (1976). Inference and missing data. Biometrika.

[39] Fieuws S, Verbeke G (2005). Evaluation of the Pairwise Approach for Fitting Joint Linear Mixed Models: A Simulation Study. Technical Report TR0527.

[40] Verbeke G, Molenberghs G (2010). Arbitrariness of models for augmented and coarse data, with emphasis on incomplete data and random effects models. Stat Model.

