# Supplementary material for: Pairwise joint modeling of clustered and high-dimensional outcomes with covariate missingness in pediatric pneumonia care
Source: Pharm Stat. Author manuscript; Available in PMC 2022 Sep 22. (PMC7613603; doi:10.1002/pst.2197)
Supplement: Appendix S1: Suplement Appendix Tables [file EMS152550-supplement-Appendix_S1__Suplement_Appendix_Tables.docx]

Table A1: Odds ratios and 95% confidence intervals under complete case analysis. Pairwise joint modelling of nine pneumonia care outcomes.

|  | **Cough** | **Difficult breathing** | **Respiratory rate** | **Oxygen saturation** | **AVPU^a^** | **Indrawing** | **Correct diagnosis** | **Correct treatment** | **Correct dose** |
| --- | --- | --- | --- | --- | --- | --- | --- | --- | --- |
| Effect | OR  (95% CI) | OR  (95% CI) | OR  (95% CI) | OR  (95% CI) | OR  (95% CI) | OR  (95% CI) | OR  (95% CI) | OR  (95% CI) | OR  (95% CI) |
| Patient’s age:12-59 months | 1.16  (1.02,1.3) | 1.35 (1.04,1.66) | 0.95 (0.75,1.15) | 0.99 (0.85,1.13) | 1.09 (0.91,1.27) | 1.27 (1.06,1.48) | 1.33 (1.05,1.61) | 1.15 (0.73,1.57) | 1.13 (0.68,1.58) |
| Patient’s gender: males | 1.02 (0.82,1.22) | 0.94 (0.76,1.12) | 0.89 (0.72,1.06) | 0.89 (0.76,1.02) | 0.86 (0.69,1.03) | 1.07 (0.89,1.25) | 0.91 (0.73,1.09) | 0.98  (0.66,1.3) | 0.96 (0.44,1.48) |
| Comorbidities: 0 | 1.32  (0.94,1.75) | 1.76 (1.25,2.17) | 1.27 (1.06,1.44) | 1.34 (1.06,1.62) | 1.49 (1.19,1.79) | 1.26  (1.05,1.47) | 0.89 (0.63,1.15) | 2.15 (1.74,2.56) | 1.96 (1.58,2.45) |
| Comorbidities :1 | 1.35  (0.96,1.74) | 1.74 (1.29,2.19) | 1.25 (1.03,1.47) | 1.25  (1.04,1.46) | 1.66 (1.46,1.86) | 1.24 (1.09,1.39) | 1.06 (0.9,1.22) | 2.13  (1.86,2.4) | 1.97 (1.65,2.23) |
| Comorbidities :2 | 1.34 (0.95,1.73) | 1.73 (1.21,2.15) | 1.21 (1.04,1.38) | 1.22 (1.03,1.41) | 1.78 (1.55,2.01) | 1.20 (1.03,1.38) | 1.10 (0.89,1.31) | 1.66 (1.29,2.03) | 1.94 (1.66,2.3) |
| Clinician’s gender: female | 0.92 (0.66,1.18) | 1.23 (0.90,1.56) | 1.08 (0.84,1.32) | 1.22 (0.98,1.45) | 1.45 (1.24,1.66) | 1.23 (1.03,1.43) | 1.17  (1,1.34) | 1.24 (0.94,1.54) | 1.32 (1.08,1.56) |
| Clinician’s cadre: MO^b^ | 1.19 (0.95,1.43) | 0.65 (0.18,1.12) | 1.58 (1.31,1.85) | 1.47  (1.24,1.7) | 1.19  (1.02,1.36) | 1.83 (1.53,2.13) | 0.94 (0.66,1.22) | 0.95 (0.71,1.19) | 0.91 (0.72,1.1) |
| Hospital workload: low | 0.82  (0.4, 1.15) | 1.54 (1.25,1.83) | 1.01 (0.64,1.38) | 0.64  (0.4,0.88) | 0.87 (0.6,1.14) | 0.25 (0.13,0.37) | 1.17 (0.94,1.4) | 1.33 (1.13,1.53) | 0.44 (0.17,0.71) |
| Malaria prevalence: low | 0.87  (0.60,1.14) | 0.73 (0.43,1.03) | 1.93 (1.40,2.46) | 0.22  (0.04,0.4) | 0.82 (0.62,0.98) | 0.89 (0.72,1.06) | 1.19 (0.96,1.42) | 0.83 (0.66,1.02) | 0.82 (0.60,1.02) |
| Time (months) | 1.24 (0.97,1.51) | 1.17 (0.95,1.39) | 1.06 (0.87,1.25) | 1.27 (1.07,1.47) | 1.10 (0.87,1.33) | 1.52 (1.2,1.84) | 1.02 (0.88,1.16) | 0.90 (0.74,1.06) | 1.04 (0.92,1.16) |
| Enhanced A&F^c^ arm | 1.01 (0.85,1.17) | 0.76 (0.39,1.13) | 0.85 (0.57,1.13) | 0.84 (0.5,1.18) | 0.78 (0.59,0.98) | 0.89 (0.63,1.15) | 0.79  (0.60,0.98) | 0.82  (0.60,1.04) | 0.94 (0.75,1.13) |
| Time× Enhanced A&F arm | 1.04  (0.78,1.3) | 1.05 (0.81,1.29) | 1.18 (1.04,1.32) | 1.26  (1.02,1.50) | 1.16 (0.87,1.45) | 1.24 (1.01,1.47) | 1.36 (1.11,1.61) | 1.32 (1.06,1.58) | 1.17 (1.02,1.32) |

AVPU^a^:-Alert, Verbal response, Pain response, Unresponsive, MO^b^:- Medical officers , A&F^c^:-Audit and feedback

Table A2: Odds ratios and the 95% confidence intervals after multiple imputation of missing covariates. Pairwise joint modelling of nine pneumonia care outcomes.

|  | **Cough** | **Difficult breathing** | **Respiratory rate** | **Oxygen saturation** | **AVPU^a^** | **Indrawing** | **Correct diagnosis** | **Correct treatment** | **Correct dose** |
| --- | --- | --- | --- | --- | --- | --- | --- | --- | --- |
| **Effect** | OR  (95% CI) | OR  (95% CI) | OR  (95% CI) | OR  (95% CI) | OR  (95% CI) | OR  (95% CI) | OR  (95% CI) | OR  (95% CI) | OR  (95% CI) |
| Patient’s age: 12-59 months | 1.15 (1.02,1.28) | 1.37 (1.1,1.64) | 0.91 (0.78,1.04) | 1.02 (0.88,1.16) | 1.11 (0.87,1.35) | 1.28  (1.10, 1.45) | 1.30 (1.05,1.55) | 1.13 (0.85,1.41) | 1.18 (0.87,1.49) |
| Patient’s gender: males | 1.04 (0.88,1.20) | 0.96 (0.82,1.10) | 0.90 (0.78,1.02) | 0.90 (0.77,1.03) | 0.87 (0.77,0.97) | 1.06  (0.92,1.20) | 0.89 (0.79,0.99) | 0.98 (0.7,1.26) | 0.99 (0.54,1.44) |
| Comorbidities: 0 | 1.33 (0.96,1.7) | 1.66 (1.28,2.04) | 1.25 1.08,1.42) | 1.40 (1.19,1.61) | 1.35 (1.19,1.51) | 1.22  (1.06,1.38) | 0.87 (0.66,1.08) | 2.09 (1.69,2.49) | 2.12 (1.41,2.83) |
| Comorbidities :1 | 1.34 (0.98,1.69) | 1.68 (1.29,2.08) | 1.23 (1.06,1.40) | 1.30 (1.12,1.48) | 1.68 (1.49,1.87) | 1.25  (1.11,1.39) | 1.03 (0.88,1.18) | 1.89 (1.68,2.10) | 1.90 (1.71,2.09) |
| Comorbidities :2 | 1.32 (0.98,1.66) | 1.69 (1.30,2.04) | 1.90  (1.07,1.31) | 1.24 (1.07,1.41) | 1.65 (1.49,1.81) | 1.22  (1.01,1.43) | 1.08 (0.9,1.26) | 1.83 (1.55,2.11) | 1.54 (1.22,1.86) |
| Clinician’s gender: female | 1.18 (0.95,1.41) | 1.25 (0.96,1.55) | 1.05 (0.89,1.21) | 1.19 (1.03,1.35) | 1.47 (1.32,1.62) | 1.24  (1.06,1.42) | 1.12  (1.0,1.24) | 1.14 (0.98,1.3) | 1.27 (1.12,1.42) |
| Clinician’s cadre: MO^b^ | 1.17 (0.96,1.38) | 0.52 (0.12,0.92) | 1.64 (1.48,1.8) | 1.45  (1.3,1.6) | 1.17 (1.02,1.32) | 1.83  (1.55,2.11) | 0.96 (0.77,1.15) | 0.91 (0.73,1.09) | 0.98 (0.79,1.17) |
| Hospital workload: low | 0.85 (0.57,1.11) | 1.44 (1.25,1.63) | 0.93 (0.69,1.17) | 0.57 (0.41,0.73) | 0.79 (0.62,0.96) | 0.24  (0.15,0.33) | 1.15 (0.97,1.33) | 1.30 (1.18,1.42) | 0.48 (0.21,0.75) |
| Malaria prevalence: low | 0.85 (0.65,1.05) | 0.70 (0.48,0.92) | 1.93 (1.49,2.35) | 0.26 (0.16,0.36) | 0.83 (0.70,0.96) | 0.89  (0.75,1.03) | 1.18 (0.98,1.38) | 0.85 (0.71,0.99) | 0.81 (0.68,0.94) |
| Time (months) | 1.22 (1.02,1.42) | 1.21  (1,1.42) | 1.04 (0.88,1.2) | 1.23 (1.08,1.38) | 0.99 (0.88,1.10) | 1.53  (1.25,1.81) | 0.99 (0.89,1.09) | 0.93 (0.81,1.05) | 0.98 (0.86,1.10) |
| Enhanced A&F^c^ arm | 0.95 (0.78,1.12) | 0.86  (0.6,1.12) | 0.82 (0.68,0.96) | 0.85  (0.60,1.10) | 0.76 (0.6,0.92) | 0.86  (0.65,1.07) | 0.74 (0.6,0.88) | 0.84 (0.65,1.03) | 0.87 (0.68,1.06) |
| Time × Enhanced A&F arm | 0.98 (0.8,1.16) | 0.96 (0.76,1.16) | 1.17 (1.06,1.28) | 1.25 (1.05,1.45) | 1.17 (0.92,1.42) | 1.26  (1.08,1.44) | 1.37 (1.12,1.62) | 1.34 (1.1,1.58) | 1.16 (1.03,1.29) |

AVPU^a^:-Alert, Verbal response, Pain response, Unresponsive, MO^b^:- Medical officers , A&F^c^:-Audit and feedback

Table A3: Odds ratios and the 95% confidence intervals under complete case analysis: Separate analysis of nine pneumonia care outcomes.

|  | **Cough** | **Difficult breathing** | **Respiratory rate** | **Oxygen saturation** | **AVPU^a^** | **Indrawing** | **Correct diagnosis** | **Correct treatment** | **Correct dose** |
| --- | --- | --- | --- | --- | --- | --- | --- | --- | --- |
| **Effect** | OR  (95% CI) | OR  (95% CI) | OR  (95% CI) | OR  (95% CI) | OR  (95% CI) | OR  (95% CI) | OR  (95% CI) | OR  (95% CI) | OR  (95% CI) |
| Patient’s age: 12-59 months | 0.90 (0.72,1.10) | 0.89  (0.66,1.12) | 0.91 (0.72,1.10) | 1.03 (0.85,1.21) | 0.74 (0.57,0.91) | 1.06  (0.89,1.23) | 1.29 (1.06,1.52) | 1.27 (1.03,1.51) | 1.17  (1.01,1.34) |
| Patient’s gender: males | 0.87 (0.68,1.06) | 0.94  (0.7,1.18) | 0.90 (0.71,1.08) | 0.87  (0.74,1.0) | 1.05 (0.79,1.31) | 1.09  (0.85,1.33) | 0.89 (0.77,1.01) | 0.98 (0.88,1.08) | 0.97 (0.84,1.10) |
| Comorbidities: 0 | 1.32 (0.9,1.74) | 1.6  (1.31,1.91) | 1.25 (1.02,1.48) | 1.21 (0.93,1.49) | 1.26 (1.04,1.52) | 1.11  (0.9,1.32) | 0.95 (0.73,1.17) | 1.23 (0.96,1.50) | 1.35 (1.09,161) |
| Comorbidities :1 | 1.34 (0.94,1.73) | 1.58  (1.34,1.92) | 1.27 (1.04,1.3) | 1.22 (0.91,1.53) | 1.27 (1.03,1.56) | 1.12  (0.94,1.28) | 1.02 (0.75,1.29) | 1.24 (0.95,1.53) | 1.39 (1.11,1.67) |
| Comorbidities :2 | 1.31 (0.92,1.70) | 1.55  (1.33,1.77) | 1.24  (1.03,1.45) | 1.21 (0.92,1.50) | 1.25 (1.05,1.51) | 1.12  (0.95,1.29) | 1.03 (0.74,1.32) | 1.26 (0.94,1.54) | 1.37  (1.13,1.61) |
| Clinician’s gender: female | 1.13 (0.88,1.38) | 0.90  (0.68,1.14) | 1.06 (0.89,1.23) | 1.16 (1.04,1.48) | 1.19 (0.95,1.43) | 1.15  (0.92,1.38) | 1.12 (0.84,1.40) | 1.19 (0.97,1.41) | 1.20 (0.93,1.47) |
| Clinician’s cadre: MO^b^ | 1.39 (1.06,1.72) | 0.76  (0.54,0.99) | 1.31  (1.08,1.54) | 1.27 (1.05,1.49) | 1.07 (0.76,1.38) | 1.25  (1.03,1.47) | 0.85 (0.65,1.05) | 0.93 (0.78,1.08) | 0.94 (0.74,1.14) |
| Hospital workload: low | 0.96 (0.75,1.17) | 1.21  (0.89,1.57) | 0.96 (0.77,1.14) | 0.76 (0.45,1.07) | 1.11 (0.77,1.45) | 0.68  (0.42,0.94) | 1.62 (1.4,1.84) | 1.37 (1.07,1.67) | 0.84  (0.63,1.05) |
| Malaria prevalence: low | 0.92 (0.7,1.14) | 0.88  (0.69,1.07) | 1.27 (1.09,1.45) | 0.88 (0.68,1.08) | 1.17 (0.93,1.41) | 0.97  (0.66,1.28) | 1.05 (0.90,1.20) | 0.91 (0.74,1.08) | 0.82 (0.60,1.04) |
| Time (months) | 0.98 (0.84,1.18) | 0.98  (0.86,1.11) | 1.03 (0.90,1.15) | 1.21 (1.13,1.29) | 1.06 (0.90,1.20) | 1.20  (1.03,1.37) | 0.98  (0.80,1.16) | 0.93 (0.76,1.08) | 1.01 (0.83,1.18) |
| Enhanced A&F^c^ arm | 0.67 (0.41,0.93) | 0.81  (0.58,1.04) | 0.86 (0.61,1.12) | 0.83 (0.63,1.03) | 1.17 (0.95,1.38) | 1.17  (1.01,1.32) | 0.95 (0.82,1.08) | 0.92 (0.71,1.11) | 0.72 (0.42,0.96) |
| Time × Enhanced A&F arm | 1.01 (0.80,1.2) | 1.10  (0.86,1.26) | 1.24 (1.04,1.40) | 1.18 (1.02,1.34) | 0.92 (0.75,1.09) | 0.95  (0.76,1.14) | 1.37 (1.17,1.57) | 1.34 (1.11,1.57) | 1.21 (1.02,1.42) |
| Random clinicians’  intercepts variance (standard error) | 2.76 (1.04) | 4.4 (2.09) | 2.08 (1.4) | 4.7 (2.2) | 3.23 (0.12) | 3.45 (1.34) | 1.06 (0.96) | 0.50 (0.71) | 0.63 (0.79) |

AVPU^a^:-Alert, Verbal response, Pain response, Unresponsive, MO^b^:- Medical officers , A&F^c^:-Audit and feedback

Table A4: Odds ratios and the 95% confidence intervals after multiple imputation of missing covariates: Separate analysis of nine pneumonia care outcomes.

|  | **Cough** | **Difficult breathing** | **Respiratory rate** | **Oxygen saturation** | **AVPU^a^** | **Indrawing** | **Correct diagnosis** | **Correct treatment** | **Correct dose** |
| --- | --- | --- | --- | --- | --- | --- | --- | --- | --- |
| **Effect** | OR  (95% CI) | OR  (95% CI) | OR  (95% CI) | OR  (95% CI) | OR  (95% CI) | OR  (95% CI) | OR  (95% CI) | OR  (95% CI) | OR  (95% CI) |
| Patient’s age: 12-59 months | 1.08 (0.91,1.25) | 1.09 (0.84,1.34) | 0.95 (0.78,1.12) | 1.04 (0.89,1.19) | 0.83 (0.66,0.96) | 1.02  (0.90,1.15) | 1.32  (1.1,1.54) | 1.25 (1.04,1.41) | 1.13 (1.01,1.25) |
| Patient’s gender: males | 0.94 (0.78,1.10) | 0.89 (0.71,1.07) | 0.94 (0.77,1.11) | 0.98 (0.85,1.11) | 1.07 (0.85,1.29) | 1.06  (0.92,1.26) | 0.86  (0.77,0.95) | 0.98 (0.9,1.06) | 0.99 (0.90,1.08) |
| Comorbidities: 0 | 1.30 (0.96,1.66) | 1.59 (1.33,1.85) | 1.22 (1.04,1.40) | 1.18 (0.95,1.41) | 1.28 (1.08,1.48) | 1.09  (0.92,1.26) | 0.97 (0.76,1.18) | 1.21 (0.98,1.43) | 1.36 (1.14,1.58) |
| Comorbidities :1 | 1.31  (0.98,1.63) | 1.56 (1.37,1.75) | 1.24 (1.05,1.43) | 1.15 (0.92,1.38) | 1.29 (1.04,1.53) | 1.07  (0.93,1.21) | 1.03  (0.8,1.26) | 1.22 (0.99,1.45) | 1.38 (1.16,1.6) |
| Comorbidities :2 | 1.32 (0.98,1.65) | 1.53 (1.38,1.68) | 1.23 (1.06,1.40) | 1.19 (0.98,1.39) | 1.27 (1.07,1.47) | 1.08  (0.94,1.22) | 1.01  (0.77,1.25) | 1.23 (0.97,1.49) | 1.35 (1.18,1.52) |
| Clinician’s gender: female | 1.08 (0.85,1.31) | 0.95 (0.71,1.19) | 1.04 (0.92,1.16) | 1.23 (1.06,1.40) | 1.17 (0.98,1.36) | 1.12  (0.94,1.30) | 1.08 (0.88,1.28) | 1.14 (0.93,1.35) | 1.12 (0.88,1.36) |
| Clinician’s cadre: MO^b^ | 1.23 (0.98,1.48) | 0.89 (0.67,1.11) | 1.29  (1.10,1.48) | 1.27 (1.06,1.48) | 1.04 (0.76,1.32) | 1.23  (1.05,1.41) | 0.92 (0.78,1.06) | 0.89 (0.74,1.04) | 0.95 (0.79,1.11) |
| Hospital workload: low | 1.01 (0.82,1.18) | 1.26 (0.95,1.57) | 0.94 (0.79,1.09) | 0.78 (0.58,0.97) | 1.15  (0.81,1.41) | 0.65  (0.41,0.89) | 1.64 (1.45,1.83) | 1.34 (1.09,1.59) | 0.81  (0. 68,0.94) |
| Malaria prevalence: low | 0.96 (0.75,1.17) | 0.89 (0.74,1.04) | 1.33 (1.17,1.49) | 0.87 (0.69,1.05) | 1.16 (0.95,1.33) | 0.88  (0.64,1.12) | 1.07  (0.96,1.17) | 0.96 (0.86,1.06) | 0.85 (0.66,1.04) |
| Time (months) | 1.01  (0.85,1.16) | 0.98  (0.89,1.10) | 1.02 (0.95,1.10) | 1.15  (1.04,1.26) | 1.09 (0.98,1.19) | 1.06  (0.95,1.17) | 0.97 (0.83,1.11) | 0.90 (0.78,1.02) | 0.98 (0.85,1.11) |
| Enhanced A&F^c^ arm | 0.77 (0.5,1.04) | 0.83  (0.60,1.03) | 0.82 (0.60,1.02) | 0.85 (0.70,1.01) | 1.15 (0.98,1.32) | 1.16  (1.01,1.30) | 0.91 (0.77,1.05) | 0.89 (0.79,0.99) | 0.67 (0.45,0.89) |
| Time × Enhanced A&F arm | 0.98 (0.82,1.18) | 1.05  (0.93,1.22) | 1.26 (1.08,1.44) | 1.17 (1.05,1.30) | 0.95 (0.80,1.15) | 1.18  (1.04,1.32) | 1.36 (1.23,1.49) | 1.31 (1.07,1.54) | 1.18 (1.05,1.31) |
| Random clinicians’  intercepts variance (standard error) | 2.23 (0.95) | 3.95 (1.78) | 1.97 (1.21) | 4.16 (1.98) | 3.04 (0.19) | 2.56 (1.12) | 1.01 (0.86) | 0.52 (0.68) | 0.57 (0. 49) |
